# Optical Fiber Vibration Signal Recognition Based on the EMD Algorithm and CNN-LSTM

**DOI:** 10.3390/s25072016

**Published:** 2025-03-23

**Authors:** Kun Li, Yao Zhen, Peng Li, Xinyue Hu, Lixia Yang

**Affiliations:** 1 School of Electronic Information Engineering, Anhui University, Hefei 230601, China; 2 8th Research Institute of China Electronics Technology Group Corporation, Hefei 230051, China

**Keywords:** distributed optical fiber vibration sensing (DAS), fiber perimeter warning, empirical mode decomposition, deep learning

## Abstract

Accurately identifying optical fiber vibration signals is crucial for ensuring the proper operation of optical fiber perimeter security warning systems. To enhance the recognition accuracy of intrusion events detected by the distributed acoustic sensing system (DAS) based on phase-sensitive optical time-domain reflectometer (φ-OTDR) technology, we propose an identification method that combines empirical mode decomposition (EMD) with convolutional neural networks (CNNs) and long short-term memory (LSTM) networks. First, the EMD algorithm decomposes the collected original optical fiber vibration signal into several intrinsic mode functions (IMFs), and the correlation coefficient between each IMF and the original signal is calculated. The signal is then reconstructed by selecting effective IMF components based on a suitable threshold. This reconstructed signal serves as the input for the network. CNN is used to extract time-series features from the vibration signal and LSTM is employed to classify the reconstructed signal. Experimental results demonstrate that this method effectively identifies three different types of vibration signals collected from a real-world environment, achieving a recognition accuracy of 97.3% for intrusion signals. This method successfully addresses the challenge of φ-OTDR pattern recognition and provides valuable insights for the development of practical engineering products.

## 1. Introduction

The DAS based on the φ-OTDR is widely used in pipeline leakage monitoring [1], perimeter security intrusion [2], rail transit safety [3], and other fields [4] because of its high sensitivity, strong anti-electromagnetic ability, relatively low cost, and ease of remote intrusion detection [5]. In recent years, extensive research has been conducted on the classification and recognition of optical fiber vibration signals [6]. For example, in 2019, Huijuan Wu et al. proposed a CNN-SVM method for φ-OTDR technology to identify vibration events along a pipeline and improve the recognition efficiency by denoising the optical fiber signal [7]. In 2021, He Meng et al. constructed a data-driven XGBoost model to classify different intrusion events. They also introduced the EMD-CIIT algorithm to denoise vibration signals, extract both time-domain and frequency-domain features, and train the model for classification [8]. Chengjin Xu et al. proposed a pattern recognition method based on combining time-frequency analysis and neural networks to solve the vibration recognition problem in optical fiber sensing systems. Experimental results show that this method greatly improves the recognition accuracy of the φ-OTDR system in a complex noise environment and can achieve real-time recognition [9]. Yuzhou Du et al. proposed an algorithm to convert one-dimensional vibration signals into grayscale images and added a channel attention mechanism to improve the recognition accuracy of vibration signals [10].

However, most conventional fiber vibration signal recognition studies focus mainly on feature extraction [11]. The accuracy of signal classification is directly affected by the type of features extracted. Since feature extraction often relies on manual experience, evaluating how well the extracted features match with the pattern recognition algorithm is challenging and lacks scientific guidance. Converting one-dimensional vibration signals into images for recognition will increase the hardware requirements.

Given the limitations of traditional feature extraction methods, deep learning approaches offer an alternative by performing end-to-end signal classification without the need for manual feature extraction [12]. These methods generally demonstrate better performance in multi-classification problems with large sample sizes [13]. Neural networks update model parameters and extract features through learning and error backpropagation using large batches of training samples, thereby completing model training effectively.

In order to avoid the influence of human participation, achieve complementary advantages of each network, and complete end-to-end signal classification and recognition of feature extraction and classification evaluation, this paper proposes a one-dimensional fiber vibration signal recognition model combining CNN and LSTM. The preprocessed vibration signal is directly used as the input of the model, and CNN is used to highlight the feature information of the vibration signal. The extracted feature information is then used as the input of the LSTM network layer, and, finally, the intelligent classification and recognition of the fiber vibration signal is realized. This method avoids the step of manually extracting signal features and does not need to occupy more hardware resources to convert the vibration signal into image processing. It has important reference value for practical engineering applications with limited hardware resources [14].

## 2. Materials and Methods

### 2.1. Empirical Mode Decomposition

The empirical mode decomposition (EMD) algorithm can adaptively decompose non-stationary time-series and nonlinear signals from a high frequency to a low frequency into the sum of N intrinsic mode functions (IMF) and a residual component [15].

(1) $$x\left(t\right)=\sum_{i=1}^{N}\;y_{i}\left(t\right)+\delta \left(t\right)$$

Wherein $y_{i}\left(t\right)$ is the IMF component obtained by decomposition, $\delta \left(t\right)$ is the residual function, and N is the number of IMFs.

The EMD algorithm first assumes that any signal is composed of a finite number of intrinsic mode functions (IMFs), and each intrinsic mode function must satisfy the following two requirements: the extreme points and zero-crossing points of the component signal must be equal or the number must differ by at most one; at any point of the IMF, the average value of the upper envelope defined by the maximum point and the lower envelope defined by the minimum point is zero.

The detailed steps of the EMD algorithm are as follows.

Step 1: Find all maximum and minimum points of the signal to be decomposed x(t).

Step 2: Fit the envelope function, perform cubic spline interpolation on the maximum points to obtain the upper envelope, and perform cubic spline interpolation on the minimum points to receive the lower envelope, which is recorded as $e_{\max}\left(t\right)$ and $e_{\min}\left(t\right)$, respectively.

Step 3: Calculate the average value of the upper and lower envelopes, $m(t)=\left(e_{\min}(t)+e_{\max}\left(t\right)\right)/2$.

Step 4: Calculate the IMF, and subtract m(t) from the original signal sequence x(t) to obtain a new signal y(t) without a low frequency.

Step 5: Determine whether the new signal satisfies the IMF condition. Otherwise, the above process is repeated. A screening threshold was introduced to determine whether the signal decomposition was completed. The ε value is usually between 0.2 and 0.3. Determine whether the SD is greater than a given threshold. If not, y(t) is a first-order IMF; otherwise, go back to the second step. The screening threshold for this experiment was set to 0.3.

(2) $$\mathrm{SD}=\frac{\sum {\left[y_{k}\left(t\right)-y_{k-1}\left(t\right)\right]}^{2}}{\sum {{[y}_{k-1}\left(t\right)]}^{2}}$$

Step 6: Determine whether the residual δ(t) is a monotonic function or a constant. If so, the EMD decomposition process ends.

Because the EMD algorithm is adaptable, the method is simple and intuitive, and there is no need to predefine the basis function, it is widely used in signal processing and analysis fields such as vibration signals, seismic signals, and electrocardiogram signals.

### 2.2. Pearson Correlation Coefficient Method

Modal aliasing is a common problem in the EMD decomposition process. When modal aliasing appears in the IMF component, it causes the IMF component to have no physical meaning and affects the subsequent signal decomposition. Therefore, the Pearson correlation coefficient (PCC) was used to calculate the correlation coefficient R and correlation index P of each IMF component and the original signal [16].

In the calculation process, *p* is the first criterion (the general threshold is 0.05); that is, when *p* > 0.05, it is directly assumed that the component is not related to the original signal. The value of R ranged between −1 and 1. When R is close to 1, the two variables are positively correlated. When R is closer to −1, it means that the two variables are more negatively correlated. When R is closer to zero, the two variables are less correlated. The calculation formulae are as follows:

(3) $$P_{x,y}=\frac{\mathrm{cov}\left(x,y\right)}{\sigma_{x}\sigma_{y}}$$

(4) $$R=\frac{1}{N-1}\sum_{j=1}^{N}\;\;\left(\frac{x_{j}-\overset{‾}{x}}{\sigma_{x}}\right)\left(\frac{y_{j}-\overset{‾}{y}}{\sigma_{y}}\right)$$

Among them, $\sigma_{x},\sigma_{y}$ represent the sample mean difference in x, y, and $\overset{‾}{x},\overset{‾}{y}$ represent the sample mean of x,y.

Therefore, the PCC calculates the *p* and R values of each decomposed IMF component and the original signal. When *p* > 0.05, the IMF component was considered invalid. When the *p* value was less than 0.05, but the R-value was less than 0.2, the IMF component was also considered invalid and discarded.

After obtaining the IMF component, the PCC of each IMF component and the original event signal were calculated. IMF components that did not meet the threshold were discarded. Because some noise still existed in the low-frequency IMF components, wavelet threshold denoising (WTD) was performed on the IMF components that satisfied the threshold conditions, and the denoised IMF components were summed to obtain the reconstructed vibration signal.

### 2.3. CNN-LSTM

#### 2.3.1. Convolutional Neural Network

The structure of the CNN mainly includes an input layer, convolution layer, pooling layer, and fully connected layer [17]. The most critical part is the convolution layer, which extracts the features of the input data through a convolution operation, then adds bias terms, and obtains a series of feature maps through the activation function.

Assuming that the lth layer of the CNN network is a convolution layer and the subsequent layer is a pooling layer, the calculation formula for the lth layer convolution is

(5) $$X_{i}^{l}=f\left(\sum_{j=1}^{m^{l-1}}\left[\;X_{j}^{l-1}\cdot w_{\mathrm{ij}}^{l}+b_{j}^{l}\right]\right)\left(i=1,2,\ldots ,m^{l}\right)$$

where $X_{i}^{l}$ is the i-th output feature of the l-th layer, $X_{j}^{l-1}$ is the j-th output feature of the (l − 1) layer feature map, $w_{\mathrm{ij}}^{l}$ is the convolution kernel weight vector between the i-th output feature of the (l − 1) layer and the j-th output feature of the (l − 1) layer feature map, and $b_{j}^{l}$ is the bias term.

This experiment uses the ReLU function as the activation function, and its mathematical expression is as follows:

(6) $$f\left(x\right)=\max\left(0,x\right)$$

After the convolution layer, a pooling layer was used to reduce the dimensions of the feature map. Common pooling operations include maximum and average pooling operations. In this study, the maximum pooling layer is selected to perform the maximum pooling operation on the feature information; that is, the feature map output by the convolution layer is pooled in each non-overlapping area of size n × n, and the maximum value in each area is selected such that the final output image is reduced n times in both dimensions.

After the input fiber vibration signal is processed by multiple convolution and pooling layers, the extracted features are classified by the fully connected layer network to obtain the final classification result.

#### 2.3.2. Long Short-Term Memory Neural Network

LSTM is a special recurrent neural network (RNN). LSTM introduces a gating mechanism that can alleviate the gradient explosion and gradient vanishing problems of the RNN during training to a certain extent; that is, the information is filtered and memorized through the forget gate, input gate, and output gate [18]. The input gate can temporarily store relevant information, the forget gate determines which information is discarded from the cell state, and the output gate determines the final output information.

The information was filtered through the forget gate, and the calculation method was as follows:

(7) $$f_{t}=\sigma \left(w_{L1}\left[h_{t-1},x_{t}\right]+b_{L1}\right)$$

where σ is the sigmoid function, $w_{L1}$ and $b_{L1}$ are weights and biases, respectively, $h_{t-1}$ is the output of the previous unit, and $x_{t}$ is the current input.

The input gate determines the new information that can be memorized by the cell unit, which is generally divided into two parts. First, the sigmoid layer determines which information must be updated, and then the tanh layer generates a new cell state ${\tilde{C}}_{t}$. When updating, the old state is multiplied by $f_{t}$, and then $\;{\tilde{C}}_{t}*i_{t}$ is added to complete the update of the cell state. The formulae are as follows:

(8) $$i_{t}=\sigma \left(w_{L2}\left[h_{t-1},x_{t}\right]+b_{L2}\right)$$

(9) $${\tilde{C}}_{t}=\tan h\left(w_{L3}\left[h_{t-1},x_{t}\right]+b_{L3}\right)$$

(10) $$C_{t}=f_{t}C_{t-1}+i_{t}{\tilde{C}}_{t}$$

where $w_{L2},w_{L3}$ and $b_{L2},b_{L3}$ are the weights and biases of the input gate and memory unit, respectively, tanh is the activation function, $f_{i}$ and $i_{t}$ are the outputs of the forget gate and input gate, respectively, $C_{t}$ is the output of the memory unit, $C_{t-1}$ is the output of the previous memory unit, and ${\tilde{C}}_{t}$ is the output of the activation function tanh.

The output gate is responsible for determining the number of outputs of the cell state at the current moment. The final output $h_{t}$ is determined by the output gate output $o_{t}$ and the memory cell output $C_{t}$; that is, the sigmoid layer is used to determine which part of the cell state will be output, and then the memory cell is processed by tanh, and the two parts are multiplied to obtain the final output result. The specific calculation is as follows:

(11) $$o_{t}=\sigma \left(w_{\mathrm{LA}}\left[h_{t-1},x_{t}\right]+b_{\mathrm{LA}}\right)$$

(12) $$h_{t}=o_{t}\tan h\left(C_{t}\right)$$

where and $w_{\mathrm{LA}}{,\;b}_{\mathrm{LA}}$ are the weight and bias, respectively, of the output gate.

#### 2.3.3. Classifier Evaluation Metrics

The evaluation indicators include the accuracy $\left(A_{\mathrm{acc}}\right)$, precision $\left(P_{\mathrm{precision}}\right)$, recall $\left(R_{\mathrm{recall}}\right)$, and $F_{1}$ score [19].

Accuracy refers to the proportion of correct classifications in the classification model relative to the total sample.

(13) $$A_{\mathrm{acc}\;}=\frac{\sum_{i=1}^{k}N_{\mathrm{TPi}}}{N}$$

Precision refers to the number of true positive examples among the samples predicted as positive examples.

(14) $$P_{\mathrm{precision}\;}=\frac{N_{\mathrm{TP}}}{N_{\mathrm{TP}}+N_{\mathrm{FP}}}$$

Recall is the ratio of positive examples in a dataset that was successfully identified.

(15) $$R_{\mathrm{recall}\;}=\frac{N_{\mathrm{TP}}}{N_{\mathrm{TP}}+N_{\mathrm{FN}}}$$

Precision and recall affect each other; therefore, the F_1 value is introduced to reconcile the precision and recall, as follows:

(16) $$F_{1}=\frac{2P_{\mathrm{precision}\;}\times R_{\mathrm{re}c\mathrm{all}\;}}{P_{\mathrm{precisison}\;}+R_{\mathrm{recall}\;}}$$

where $N_{\mathrm{TP}}$ represents the number of correctly identified events A, $N_{\mathrm{FP}}$ represents the number of other events identified as A, $N_{\mathrm{FN}}$ represents the number of events identified as other events, k represents the number of event classifications, and N is the total number of samples.

### 2.4. EMD-CNN-LSTM Model

Based on the EMD-CNN-LSTM model, this study proposed a complete modeling process to make predictions and evaluate the corresponding performance [11]. The steps are as follows:

Step 1: Obtain the vibration signals, standardize them, and divide them into frames (each frame of the sample vibration signals contains 1024 data points). Construct a dataset, perform EMD on the sorted data, use PCC to remove irrelevant IMF components, perform wavelet threshold denoising on the effective components, superimpose the denoised effective components, and reconstruct the vibration signal. Then, divide the training set, validation set, and test set according to the ratio 7:1:2.

Step 2: Input the training set into the model for parameter learning and use the gradient descent method to continuously update the weights and biases. The validation set was used to evaluate the model performance, fine-tune the model parameters according to the validation results, and further optimize the model effect. Determine whether the training times m of the network have reached the preset number of iterations N. If not, return to the model training stage to continue training; 

Step 3: After completing the training stage, the model testing stage is entered, the test set is used to perform performance testing on the trained model, and the model evaluation indicators (classification accuracy, precision, recall rate, and F1 score) are calculated, and the test results are output.

**Figure 1:**
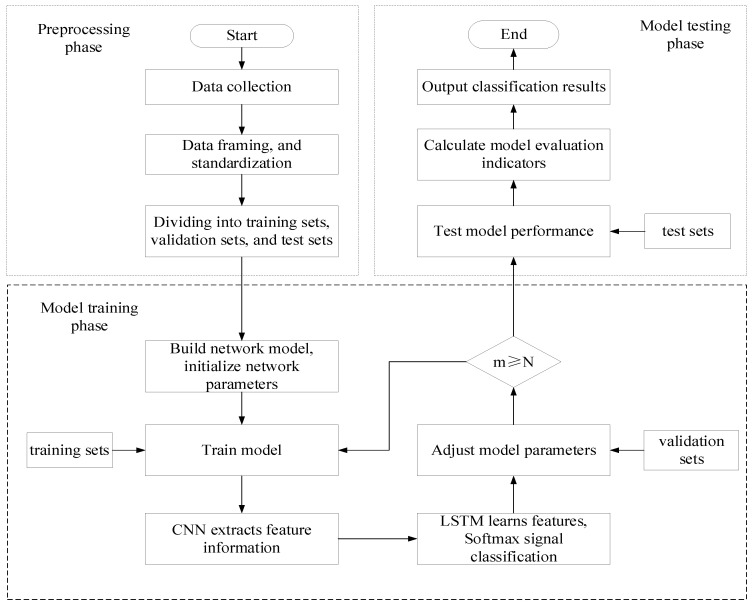
Research flow of this study.

Figure 1 illustrates the research flow of the study.

## 3. Results

### 3.1. Experiment Setup

**Figure 2:**
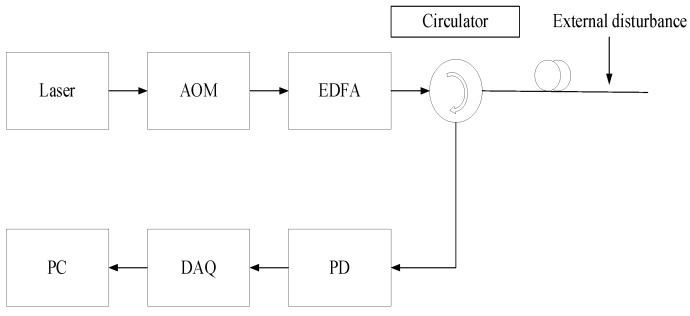
The Φ-OTDR system for vibration detection.

The working principle of the φ-OTDR distributed optical fiber vibration sensing system is shown in Figure 2. It uses a narrow linewidth laser as a light source to output continuous laser light. It uses an acoustic–optic modulator (AOM) to modulate the laser light emitted by the laser into an optical pulse with a certain pulse width and frequency. Then, the erbium-doped fiber amplifier (EDFA) amplifies the modulated optical pulse, which is injected into the sensing optical fiber through a circulator for transmission. Rayleigh scattering occurs during the transmission of the optical pulse signal along the optical cable, and its backward Rayleigh scattered optical signal returns along the optical fiber and is received by the circulator.

Under ideal conditions, the intensity of the backward Rayleigh scattered signal is only linearly related to the pulse width. When the light source is stable and no disturbance occurs, the intensity of the signal returned at each position of the optical fiber remains stable; conversely, when the optical fiber is disturbed by an external event, the phase of the backward Rayleigh scattered light at that position changes due to the elastic–optic effect (the manufacturing process of the optical fiber and the change in the refractive index caused by bending). The effect signal of the external disturbance on the optical fiber is obtained by phase demodulation. The demodulated optical signal is converted into an electrical signal by a photodetector (PD), which is then collected by a data acquisition card (DAQ). After preprocessing on the PC side, different types of vibration signals are detected and identified [20].

The optical fiber vibration signal acquisition system in this experiment uses a continuous laser with a wavelength of 1550 nm and a linewidth of 5 kHz as the light source. An acoustic–optic modulator (AOM) modulates the laser into a pulse sequence with a pulse width of 200 ns. The optical pulses are then amplified by an erbium-doped fiber amplifier (EDFA) and injected into a standard G652 single-mode sensing optical fiber through a circulator. When the optical fiber is disturbed by external events, the Rayleigh backscattered light returns to the photodetector (PD) via the circulator, where it is converted into an electrical signal. This signal is collected by a data acquisition card (DAQ) and subsequently uploaded to a computer for processing and classification.

**Figure 3:**
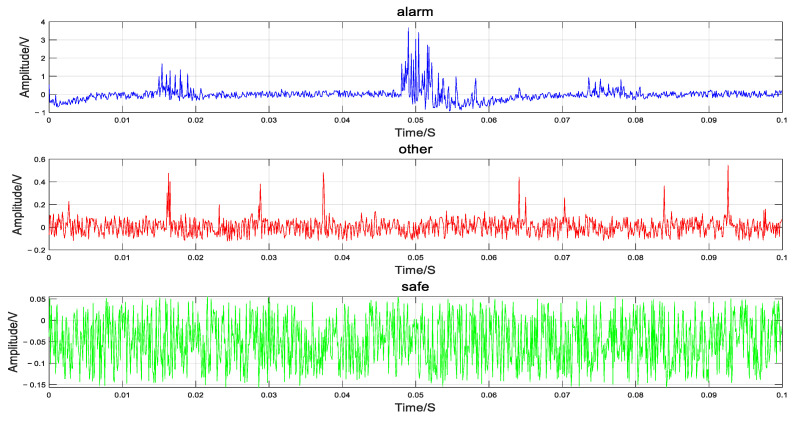
Three types of signals in the time domain.

Three types of events, including human knocking (marked as the Alarm signal in the experiment), natural noise (such as light rain, wind, etc., marked as the Other signal in the experiment), and background noise (marked as the Safe signal in the experiment), were selected as identification targets. Each type of event was collected at different times and locations, and the signal sampling rate was 10 k. The collected signals were subsequently framed and processed. Each frame of the sample vibration signal contains 1000 data points. To demonstrate the characteristics of the three types of signals, one-dimensional samples after difference processing are shown in Figure 3.

By analyzing the time-domain waveform characteristics of the three signals, it can be seen that the Alarm signal has obvious amplitude changes, and the maximum amplitude is much higher than the other two types of signals. Since the Other signal comes from the sound of wind in nature, there are some small floating and irregular amplitude changes in its time-domain waveform, and the amplitude is smaller than that of the Alarm signal. For the Safe signal, its amplitude is small, relatively stable, and presents a standard sawtooth waveform, which is significantly different from the Alarm signal waveform, mostly from the background noise in the environment and the internal noise of the components.

### 3.2. Signal Preprocessing

After collecting the vibration signals of the three types of events using a distributed optical fiber sensing system based on φ-OTDR, owing to the high sensitivity of the DAS system, the collected vibration signal data are easily affected by the environment and the internal noise of the collection system, and the signal needs to be preprocessed [21]. The preprocessing methods adopted in this experiment mainly included signal framing, EMD decomposition, IMF component denoising, and signal reconstruction.

**Figure 4:**
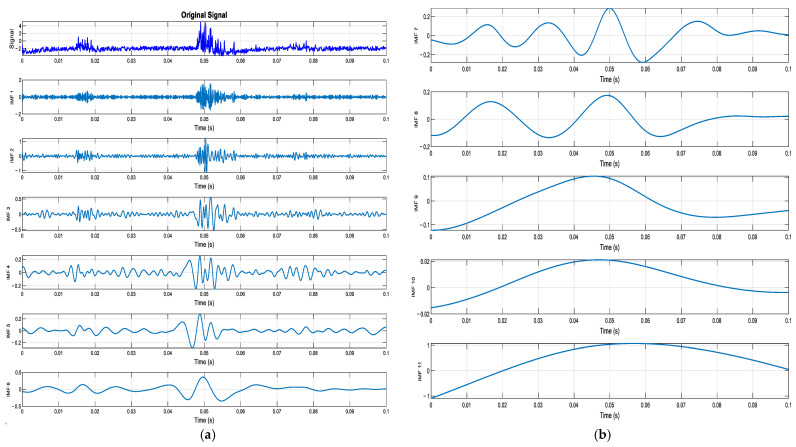
Decomposed IMF component time-domain plot. (**a**) IMF1-IMF6; (**b**) IMF7-IMF12.

The three types of vibration signals collected are framed, and then the EMD is decomposed. The frame length of each sample was approximately 0.1 s. Taking the Alarm signal as an example, the IMF component of the signal after EMD is shown in Figure 4.

**Figure 5:**
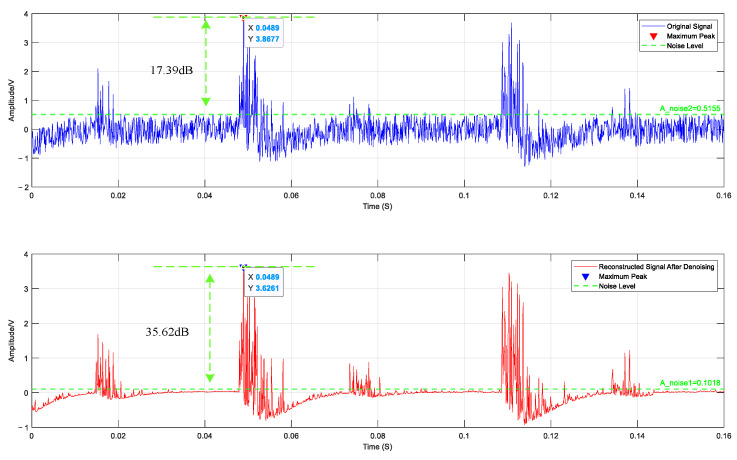
Reconstructed signal comparison chart.

After obtaining the IMF component, the Pearson correlation coefficient method was used to screen the effective IMF components of the three vibration signals. The effective IMF components were denoised by the wavelet threshold to reduce the influence of noise. Finally, the effective IMF components after denoising are summed separately to obtain the reconstructed vibration signal. Using the Alarm signal as an example, the reconstructed signal is shown in Figure 5.

The signal-to-noise ratio (SNR) is a metric used to measure the ratio of pure signal-to-noise energy. For fiber optic sensing signals, the higher the SNR, the less noise is mixed in the signal. However, under the current circumstances, the φ-OTDR system cannot collect pure signals, that is, it is impossible to obtain the SNR of the measured signal. Therefore, this experiment defines the SNR as the voltage ratio between the peak value at the signal disturbance position and the noise fluctuation range.

(17) $$SNR=20\;\log_{10}\frac{A_{peak}}{A_{noise}}$$

In Figure 5, the SNR of the original Alarm signal collected is 17.39 dB. After using the above denoising algorithm, the SNR of the original signal is increased to 35.62 dB. It can be seen from Figure 5 that the algorithm retains the overall contours of the signal’s time-domain waveform, such as the peaks and troughs, and the high-frequency information at the disturbance position is also maintained. At the same time, the noise at other positions is suppressed, and the signal’s SNR is improved [22].

### 3.3. Model Construction

Traditional machine learning requires manual experience to extract the features of the vibration signals for classification and identification. Convolutional neural networks are widely used in time-series data analysis owing to their powerful feature extraction characteristics and the absence of manual feature extraction. They captured the time dependencies in the fiber vibration data by extracting the relationships in the spatial structure of the multidimensional time-series data. The memory units in LSTM make neural networks more suitable for time-series analysis and modeling, and layer-by-layer adaptive feature extraction gives the network stronger learning capabilities to obtain information more effectively in a time series. The CNN-LSTM hybrid model can combine the advantages of CNN and LSTM, enabling the model to process fiber vibration data better and perform identification and classification [23].

**Figure 6:**
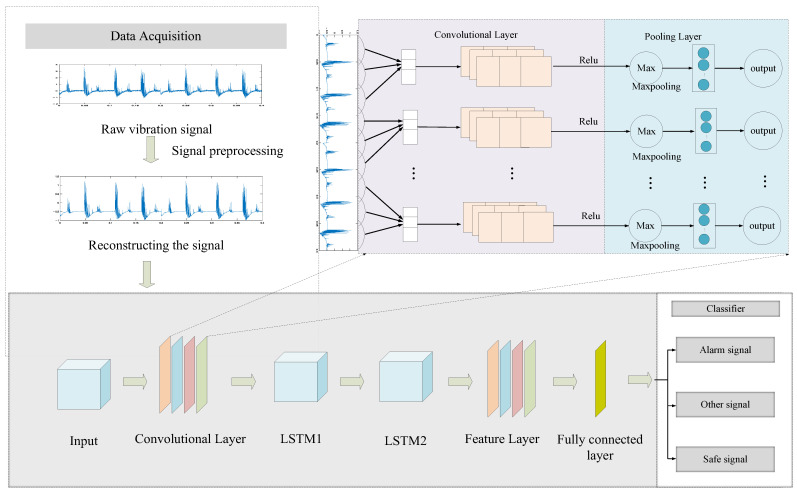
The architecture of the CNN-LSTM model.

The network structure of the optical fiber vibration signal classification model used in this experiment is shown in Figure 6. The model mainly consists of a signal input layer, CNN convolution layer, pooling layer, LSTM layer, and classification output layer. The one-dimensional optical fiber vibration signal was standardized, the EMD algorithm was used to remove the noise in the high-frequency component, and it was divided into signal segments of fixed lengths (the signal in this experiment intercepts 1024 signal points). The data sample was constructed, with 70% divided into a training set, 10% as a validation set, and 20% as a test set. The input signal was input into the CNN convolution layer to extract the features of the three different signals adaptively. The extracted features were then reduced in dimension through the maximum pooling operation, effectively reducing data redundancy and retaining key feature information. After dimensionality reduction, the feature data were then input into the LSTM layer, and its ability to model time-series dependencies was used to train the neural network.

The deep neural network used in this paper includes three convolutional layers, three pooling layers, and two LSTM layers. The network parameters of the convolutional layer are shown in Table 1.

The first convolutional layer has 64 convolution kernels, the convolution kernel size is 3, and the stride is 1. The sliding window size of the first pooling layer is two, and the sliding step is two. The second convolutional layer has 128 convolution kernels, the convolution kernel size is 3, and the stride is 1. The sliding window size of the second pooling layer is two, and the sliding step is two. The third convolutional layer has 256 convolution kernels, the convolution kernel size is 3, and the stride is 1. The sliding window size of the third pooling layer is two, and the sliding step is two. The deep features of the signal are extracted through the three-layer convolutional neural network, and then the maximum pooling operation is used to reduce the dimension of the data.

The network parameters of the LSTM layer are shown in Table 2.

After passing through the CNN layer, a linear layer with 256 outputs is used to construct the input of the LSTM layer. The first layer contains 256 hidden units and the second layer contains 128 hidden units. The first LSTM layer receives the 256-dimensional CNN-extracted features as the input and performs temporal modeling on the sequence data. The second LSTM layer further processes the temporal information, and, finally, only the last time step output of the LSTM is used for classification through the fully connected layer.

### 3.4. Experimental Results

A two-layer LSTM model and a CNN model were used to test the performance of the CNN-LSTM model for intelligent recognition and classification on the same preprocessed vibration signal dataset. The convolutional neural network model built in this experiment referred to the network configuration in the VGG model, using eight convolutional layers and three fully connected layers. The calculation results above are shown in Table 3.

It can be seen that the average accuracy of the CNN-LSTM model proposed in this paper is 97.3%, which is higher than the recognition accuracy of only using the two-layer LSTM and the VGG model.

For fiber optic perimeter security, the most important thing is to accurately identify the Alarm signal. The CNN-LSTM network is superior to other models in terms of the accuracy, recall rate, and F1 score for the Alarm signal recognition.

All of the models input preprocessed vibration data. The model’s initial learning rate was 0.001. The batch size was 32. Each time, 50 epochs were calculated, and the average of the results of five calculations was taken as the experimental result.

**Figure 7:**
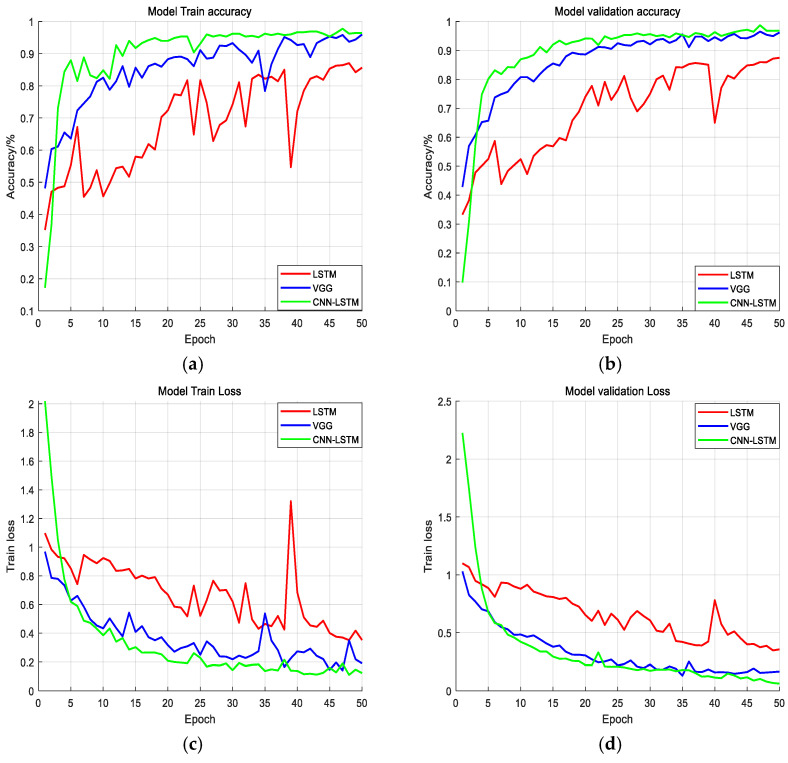
The training loss and accuracy curves for different models. (**a**) The average accuracy of the training set; (**b**) The average accuracy of the validation set; (**c**) The training set loss function; (**d**) The validation set loss function.

Figure 7 shows the accuracy and training loss of different models on the training set and test set. After 15 epochs, the accuracy of the CNN-LSTM network model stabilized at 90%, which is better than the LSTM and VGG models.

In terms of training loss, the CNN-LSTM network converges faster than the other two models. After 35 epochs, the training loss hovers around 0.2. As can be seen from the figure, the classification ability based on the CNN-LSTM network is better than that of the traditional CNN network and LSTM network.

It is proved that the CNN-LSTM network model based on the EMD decomposition algorithm proposed in this paper can effectively identifies three different types of optical fiber vibration signals, and the model has a good generalization ability.

### 3.5. Discussion

To evaluate the performance of the CNN-LSTM vibration signal classification model based on the EMD algorithm, this paper compares it with the CNN-LSTM algorithm based on the combination of time-frequency features proposed in the literature [24]. According to the method proposed in this paper, the time-domain curve of the acquired signal and its DWT (discrete wavelet transform) and STFT (short-time Fourier transform) are used as network inputs. After completing the feature extraction of the three parts, the established goal of pattern recognition is achieved by integrating the features. In the STFT, the window function selects the Hanning window, the window length is set to 64, the window overlap length is 8, the FFT point number is 512, and the sampling rate is 1000 Hz. In the DWT, the db4 wavelet basis is used to perform a four-layer discrete wavelet decomposition (DWT) on the signal.

To more accurately compare the performance of the algorithm proposed in this paper with the above algorithms, we built the neural network model in the above reference and verified it using our private dataset.

Table 4 shows the recognition results of the two algorithms for three events (the CNN-LSTM network based on the EMD algorithm proposed in this experiment is marked as Net1, and the CNN-LSTM network with time-frequency feature fusion used in the literature is marked as Net2). The input to Net1 is the vibration data reconstructed through the EMD. The time-domain curve of the collected signal, along with its DWT and STFT, serve as the input to Net2. The initial learning rate for both models is 0.001, with a batch size of 32. Each computation is performed for 50 epochs, and the average of the results from five computations is taken as the experimental outcome.

As shown in Table 4, for the fiber vibration signal data used in this experiment, Net1 outperforms Net2 in the classification of the three different vibration signals. Net1 significantly outperforms Net2 in recognizing Alarm signals, and its average accuracy is also higher than that of Net2.

## 4. Conclusions

Aiming at the problem of φ-OTDR pattern recognition, this paper proposes a hybrid neural network based on EMD decomposition and CNN-LSTM as the main framework. The original vibration signal is decomposed into a series of IMFs by combining the EMD, and the irrelevant components are filtered out by PCC, and then the effective components are filtered and the signal is reconstructed. Combining the advantages of the CNN and LSTM networks, the signal features are adaptively extracted from the preprocessed data to complete the intelligent classification of optical fiber vibration signals.

The experimental results demonstrate that the CNN-LSTM network based on the EMD algorithm used in this study exhibits excellent performance. It surpasses the single-layer LSTM network, the typical CNN network, and the CNN-LSTM network based on combined time-frequency feature extraction in terms of the recognition accuracy. For the three types of signals—Alarm signals, natural noise signals, and undisturbed signals—the proposed EMD-based CNN-LSTM achieves a recognition accuracy of 97.3% on the test device, accomplishing the predetermined goal of fiber vibration signal classification.

In future work, we will continue to explore how to further improve the classification and recognition of fiber vibration sensing signals by integrating signal processing techniques and optimizing the network architecture.

## Figures and Tables

**Table 1:** CNN layers model parameters.

Layers	Filters	Kernel Size	Stride
Conv1	64	3	1
Pool1	64	2	2
Conv2	128	3	1
Pool2	128	2	2
Conv3	256	3	1
Pool3	256	2	2

**Table 2:** LSTM layer parameters.

Layers	Input Size	Hidden Size
LSTM1	256	256
LSTM2	256	128

**Table 3:** Performance of our CNN-LSTM model and two other methods.

Model	Event Type	Precision	Recall	F1 Score
	Alarm	0.9565	0.7521	0.8421
LSTM	Other	0.7379	0.8352	0.7835
$A_{\mathrm{acc}\;}=0.9562$	Safe	0.8800	0.9821	0.9283
	Alarm	0.9636	0.9060	0.9339
VGG	Other	0.8866	0.9247	0.9053
$A_{\mathrm{acc}\;}=0.9406$	Safe	0.9646	0.9909	0.9776
	Alarm	1.0000	0.9262	0.9617
CNN-LSTM	Other	0.9143	0.9897	0.9505
$A_{\mathrm{acc}\;}=0.9732$	Safe	0.9804	0.9901	0.9852

**Table 4:** The comparison of the identification results of the three events of two algorithms at three locations.

Model	Identification Rates (%)			
	Alarm	Other	Safe	Average
Net1	95.62	98.87	97.47	97.32
Net2	89.95	95.68	95.11	93.58

## Data Availability

The data used for the development of this system have been obtained within the framework of the project. At the moment, they are not available online.
